# Supplementary material for: Hypertension in African Populations: Review and Computational Insights
Source: Genes (Basel). 2021 Apr 6;12(4):532. doi: 10.3390/genes12040532 (PMC8067483; doi:10.3390/genes12040532)
Supplement: Supplementary file 1 [file genes-12-00532-s001.pdf]

Supplementary Data

Table *S1*. Publication Search

Search	African countries
1	‘‘Africa’’ OR Africa* OR Algeria OR Angola OR Benin OR Botswana OR ‘‘Burkina Faso’’ OR Burundi OR Cameroon OR ‘‘Canary Islands’’ OR ‘‘Cape Verde’’ OR ‘‘Central African Republic’’ OR Chad OR Comoros OR Congo OR ‘‘Democratic Republic of Congo’’ OR Djibouti OR Egypt OR ‘‘Equatorial Guinea’’ OR Eritrea OR eSwatini OR Ethiopia OR Gabon OR Gambia OR Ghana OR Guinea OR ‘‘Guinea Bissau’’ OR ‘‘Ivory Coast’’ OR ‘‘Cote d’Ivoire’’ OR Jamahiriya OR Jamahiriya OR Kenya OR Lesotho OR Liberia OR Libya OR Libya OR Madagascar OR Malawi OR Mali OR Mauritania OR Mauritius OR Mayotte OR Morocco OR Mozambique OR Mozambique OR Namibia OR Niger OR Nigeria OR Principe OR Reunion OR Rwanda OR ‘‘Sao Tome’’ OR Senegal OR Seychelles OR ‘‘Sierra Leone’’ OR Somalia OR ‘‘South Africa’’ OR ‘‘St Helena’’ OR "South Sudan" OR Sudan OR Swaziland OR Tanzania OR Togo OR Tunisia OR Uganda OR ‘‘Western Sahara’’ OR Zaire OR Zambia OR Zimbabwe OR ‘‘Central Africa’’ OR ‘‘Central African’’ OR ‘‘West Africa’’ OR ‘‘West African’’ OR ‘‘Western Africa’’ OR ‘‘Western African’’ OR ‘‘East Africa’’ OR ‘‘East African’’ OR ‘‘Eastern Africa’’ OR ‘‘Eastern African’’ OR ‘‘North Africa’’ OR ‘‘North African’’ OR ‘‘Northern Africa’’ OR ‘‘Northern African’’ OR ‘‘South African’’ OR ‘‘Southern Africa’’ OR ‘‘Southern African’’ OR ‘‘sub Saharan Africa’’ OR ‘‘sub Saharan African’’ OR ‘‘sub-Saharan Africa’’ OR ‘‘sub-Saharan African’’
2	Uncontrolled Hypertension vs Drugs
	"resistant Hypertension " OR "uncontrolled Hypertension " AND hypertens* OR "high blood pressure" AND pharma* OR Mutat* OR SNP* OR "single nucleotide polymorphism" OR "sequence variants" OR varia* OR "genetic marker*" OR polymorph* OR gene* OR pharmacogenomic* AND Prazosin OR Alpha-Blocker OR Spironolactone OR potassium-sparing OR Amlodipine OR "Amloc" OR Calcium Channel Blocker OR "CCB Blockers" OR Hydralazine OR Vasodilator OR Atenolol OR Metoprolol OR Propranolol OR Bisoprolol OR Labetalol OR Carvedilol OR Beta-Blocker OR Captopril OR "angiotensin converting enzyme inhibitors" OR "ACE inhibitors" OR Enalapril OR Diuretic OR thiazide OR "loop" OR Furosemide OR "Lasix" OR Hydrocholorothiazide OR "HCTZ"

The list of 53 high priority genes has been used in the *in silico* analysis to identify potential drug targets for HTN therapy.

***Table S2*:** The list of the prioritised 53 genes used for the *in silico* analysis.

Genes			
AGT	*PLEKHA7*	*CLCNKB*	*PR3*
ACE	*JAG1*	*SCNN1B*	*HFE*
AGTR1	*FGF5*	*ADD1*	*BAG6*
NOS3	*EBF1*	*SUB1*	*CACNB2*
MTHFR	*STK39*	*CEP83*	*PLCE1*
ATP2B1	*CDKAL1*	*IGFBP3*	*CAND1*
APOA5	*IGF2BP2*	*CHIC2*	*ARHGAP42*
ANP	*TH*	*AGTR2*	*FES*
CYP2C8	*B2*	*MOV10*	*GOSR2*
GNB3	*CYYP11B2*	*SLC39A8*	*ZNF831*
CNNM2	*LEP*	*GUCY1A1N*	*ULK4*
CABCOCO1	*SH2B3*	*TBX5*	*CSK*
ZNF652	*CPS1*	*SLC4A7*	*MECOM*
ADRB2			

The list of HTN drugs was obtained from the FDA's office of women's health. These were used to generate the gene-drug interaction network.

***Table S3*:** The list of FDA drugs and their grouped according to their action mechanism.

ACE inhibitors	Beta Blockers	Calcium Channel Blockers	Peripherally Acting Alpha-Adrenergic Blockers

Benazepril	Bisoprolol	Felodipine	Doxazosin
Enalapril	Nadolol	Nisoldipine	Phenoxybenzamine
Enalaprilat	Acebutolol	Verapamil	Prazosin
Fosinopril	Labetalol	Nicardipine	Terazosin
Lisinopril	Betaxolol	Nifedipine	
Lisonopril	Penbutolol	Nimodipine	
Moexipril	Pindolol	Amloldipine	
Perindopril	Metoprolol	Clevidipine	
Quinapril	Nebivolol	Diltiazem	
Ramipril	Timolol	Isradipine	
Trandolapril	Carediol		
	Propranolol		

Renin Inhibitors	Vasodilators	Angiotensin II Antagonists	Centrally Acting Alpha Adrenergics	Diuretics
Aliskiren	Hydralazine	Azilsartan	Clonidine	Cholrothalidone
	Minoxidil	Candesartan	Guanfacine	Metolazone
		Eprosartan		Spironolactone
		Irbesartan		Torsemide
		Losartan		Chlorothiazide
		Olmesartan		Methyclothiazide
		Telmisratan		Hydrochlorothiazide
		Valsartan		Furosemide
				Indamide
				Hydroflumethiazide

***Table S4*:** Drugs associated with genes that are part of the co-expressed gene clusters.

Gene Cluster 1	Drugs
AGTR1	Telmisartan, Enalaprilat, Perindopril, Candesartan, Irbesartan, Eprosartan, Losartan, Olmesartan, Valsartan, Benazepril

AGT	Carvedilol, Benazepril, Enalapril, Propranolol, Pindolol, Lisopril, Atenolol, Labetalol, Valsartan, Losartan, Olmesartan, Spironolactone, Irbesartan, Candesartan, Amlodipine, Perindopril, Enalaprilat, Furosemide
ACE	Moexipril, Trandolapril, Fosinopril, Carvedilol, Quinapril, Ramipril, Perindopril, Enalaprilat, Benazepril, Enalapril, Lisinopril, Metoprolol
Gene Cluster 2	
IGF2BP2	Hydralazine
Gene Cluster 3	
ADRB2	Metoprolol, Penbutolol, Timolol, Bisoprolol, Nadolol, Betaxolol, Phenoxybenzamine, Carvedilol, Benazepril, Propranolol, Labetalol, Lisinopril, Pindolol, Atenolol, Acebutolol
